# A 3-circular RNA signature as a noninvasive biomarker for diagnosis of colorectal cancer

**DOI:** 10.1186/s12935-019-0995-7

**Published:** 2019-11-04

**Authors:** Dao-xiong Ye, Si-si Wang, Ying Huang, Pan Chi

**Affiliations:** Department of General Surgery, Fujian Medical University Union Hospital, No.29 Xinquan Road, Fuzhou, 350001 China

**Keywords:** circRNAs, Diagnostic signature, ROC curve, Biomarker

## Abstract

**Background:**

Circular RNAs (circRNAs), a novel type of noncoding RNAs, play critical roles in the initiation and progression of cancer. Emerging studies also shows that circRNAs may function as potential markers for cancer diagnosis and treatment. However, the diagnostic value of circRNAs in colorectal cancer (CRC) remains need to be unearthed.

**Methods:**

CircRNA microarray was performed to detect the differentially expressed circRNAs in eight plasma samples, including four colorectal cancer (CRC) and four normal samples. Besides, the results of microarray were validated by quantitative real-time polymerase chain reaction (qRT-PCR). Moreover, ROC curve evaluation was performed to calculate the diagnostic value of significantly dysregulated circRNAs. In order to predict the potential mechanism of the significant circRNAs, circRNA–miRNA–mRNA network was constructed based on the TargetScan, miRTarBase and MIRDB database, as well as CircInteractome online software. Furthermore, Gene Ontology (GO) and Kyoto Encyclopedia of Genes and Genomes (KEGG) pathway enrichment analyses were performed to further predict the function of meaningful circRNAs.

**Results:**

Totally three differentially expressed circRNAs were identified in CRC plasma compared to normal plasma by circRNA microarray analysis, and the results was validated by qRT-PCR. Hsa_circ_0082182, hsa_circ_0000370 and hsa_circ_0035445 were identified and ROC curves analysis was used to calculate the single and joint diagnostic value. Furthermore, GO and KEGG analyses revealed that functions were mainly cancer-related, which indicated that the circRNAs were meaningfully associated with CRC cell proliferation and metastasis.

**Conclusion:**

In conclusion, we have identified three circRNAs that are dysregulated in CRC plasma, including hsa_circ_0082182, hsa_circ_0000370 and hsa_circ_0035445. ROC curves showed that these circRNAs might have diagnostic value for colorectal cancer. Furthermore, bioinformatics analysis indicated that the above-mentioned circRNAs might be involved in the development of CRC.

## Background

Colorectal cancer (CRC) was one of the most common malignancies arising from the digestive system, acting as a major reason for cancer-related death [1]. Despite the early diagnosis and treatment of CRC developed in recent years, the 5-year survival rate for colorectal cancer is still not satisfactory, mainly because most patients are diagnosed with distant metastasis or at an advanced stage [2]. Although some traditional methods, such as imaging and hematological examinations were already used in practice, they were limited by their low sensitivity and specificity. Therefore, in the field of early diagnosis, novel biomarkers of CRC with high sensitivity and specificity are eagerly needed.

Circular RNAs (circRNAs), which are a type of RNAs closely looped with no accessible ends, are the downstream products of precursor mRNA back-splicing of a huge amount of eukaryotic genes [3]. CircRNAs display a higher cell and tissue specificity, and their closed structure is more stable than lncRNAs and miRNAs, suggesting that they may serve as potential molecular biomarkers for cancer diagnosis [4]. Now, several circRNAs were stated being able to play significant roles in CRC initiation and progression. For instance, Li et al. investigated the differentially expressed circRNAs in colorectal cancer by RNA sequencing, and they found circDDX17 was down-regulated and may act as a tumor suppressor in CRC. In addition, Jin et al. indicated that hsa_circ_0136666 was significantly up-regulated by qRT-PCR validation, and it could promote the invasion and proliferation of CRC [5, 6]. Notably, accumulating evidence indicated that circRNAs could affect tumor biology by modulating the gene transcription and translation. Except for the study of the basic experiments, Li et al. [7] also indicated that circVAPA was upregulated in CRC tissues and plasma, which could serve as a non-invasive biomarker for CRC early diagnosis.

In our study, considering the low sensitivity and specificity of traditional tumor biomarkers, we aimed to investigate novel molecular biomarker for CRC diagnosis. Since the circRNAs were proved to be more stable than linear RNAs, we chose circRNAs as the research object. circRNA microarray was performed to detect the differentially expressed circRNAs in CRC patients’ plasma. For investigating the diagnostic value of circRNAs, we chose patients from different staging and the specificity and sensitivity of circRNAs were calculated by ROC curves. Besides, bioinformatics analysis was used to predict the downstream miRNAs and mRNAs to indicate the potential mechanism of the significant circRNAs. In short, we studied the circRNAs in different ways and considered the chosen circRNAs may serve as promising biomarkers in CRC clinical application.

## Methods

### Patients and samples

Peripheral blood of 156 patients with CRC was collected at the Fujian Medical University Union Hospital between March and December 2018 for plasma isolation. The assayed patients were in different CRC TNM stages, of which 66, 33, 32 and 25 patients were in stage I, II, III, and IV, respectively. Histological examination was the standard for the confirmation of diagnosis of each patient. None of the patients was treated with radiotherapy or chemotherapy before plasma collection or had a previous medical history (PMH) of other kind of cancers or metastatic cancer from other sites. We collected blood from a patient before and on the 5th day after surgery so that the plasma samples (n = 45) were obtained preoperatively and postoperatively in pair. Based the age and gender, we individually matched the 66 healthy controls with no PMH of cancer to the CRC cases. This study was approved by the Ethics Committee of Fujian Medical University Union Hospital (2017KY088), and all patients or their guardians signed the consent form.

### Cell culture

The CRC cell lines-HCT116, SW480, SW620 and normal cell line-NCM460 were bought from Procell Life Science & Technology Co., Ltd. (Wuhan, China) and short tandem repeat (STR) profiling was applied to confirm. All the cell lines were cultured in DMEM (Gibco) with 10% fetal bovine serum (FBS) and 1% penicillin/streptomycin supplied. Then, the cell lines were grown with 5% CO_2_ at 37 °C in humidified air.

### Identification of aberrant plasma circRNAs

The Arraystar human circular RNAs array chips (Arraystar Human circRNAs chip; AS-S-CR-H-V2.0, ArrayStar Inc., Rockville, MD, USA), which contains 13, 617 circRNAs and 5261 probes for circRNAs specific splicing sites were applied. After hybridization, eight plasma samples (four CRC and four normal samples) were examined on the microarray chips. Edge R was used to normalize the microarray data and find the differentially expressed circRNAs in CRC plasma (Fold change ≧ 2.0, P < 0.05). Then, the cancer-specific circRNA-database (CSCD) and CircBase was used to identify the CRC-specific circRNAs and the details of them [8, 9]. Finally, a heatmap was performed to visualize the circRNAs and their structures were analyzed on the base of CSCD.

### Isolation and reverse transcription of RNAs

TRIzol™ LS Reagent (Invitrogen, Carlsbad, CA, USA) was applied to extract the total RNA from the plasma based on the manufacturer’s instructions. Also, the total RNA from the colorectal cancer cell lines-HCT116, SW480, SW620 and normal cell line-NCM460 was extracted with the TRIzol Reagent (Invitrogen). Then, the concentration and purity of the RNA were measured by the NanoDrop Lite spectrophotometer (Thermo Scientific). The PrimeScript™ RT reagent kit (#RR037A v.0610, TaKaRa Biotechnology Inc., Dalian, China) were used for circRNA reverse transcription. In short, 20 μL final volume of cDNA was obtained by reverse transcribing a 1000 ng total RNA with random primers.

### qRT-PCR

We used the TB Green™ Pre-mix Ex Taq™ II (TaKaRa) running on the Applied Biosystems StepOnePlus Real-Time PCR System (Thermo Fisher Scientific) to perform qRT-PCR array. 95 °C for 30 s, followed by 40 cycles at 95 °C for 5 s, and 60 °C for 30 s was used as the PCR condition. At the end of amplification, melting curves were generated and the specificity of the PCR products was promised. Normally, the relative expression levels of the circRNAs was calculated by the 2^−ΔΔCT^ method and glyceraldehyde 3-phosphate dehydrogenase (GAPDH) was served as a reference gene. Three independent experiments were repeated in the qRT-PCR assay. The divergent primers for these circRNAs were designed by Primer 6.0 software.

### CeRNA network analysis and function annotation

The miRNA sponge was known as a function of circRNAs. Several bioinformatics analyses were performed to study the interactions between circRNAs and miRNAs. Circinteractome, which based on the Targetscan algorithm, was an online software to predict the binding sites of circRNAs and miRNAs [8]. Additionally, the interaction between miRNA and mRNA was analyzed based on the miRTarBase, TargetScan, and miRDB database [9–11]. Thus, ceRNA (competing endogenous RNA) was constructed and the visualization of circRNA–miRNA–mRNA network was conducted by the Cytoscape software [12]. To further study the function of meaningful circRNAs, the potential downstream mRNAs were taken together for Gene Ontology (GO) and Kyoto Encyclopedia of Genes and Genomes (KEGG) pathway analyses. The mRNAs were used as input profile. The critical results of enrichment were accepted at a threshold ≥ 2 gene counts and a P < 0.05.

### Statistical analysis

The nonparametric Mann–Whitney U-test was applied to compare the differences between normal and CRC plasma groups. Wilcoxon matched-pairs signed-rank test or a paired t-test was used to evaluate the circRNA expression between pre-operative and post-operative groups. The correlation between circRNA expression and clinicopathological factors in CRC was analyzed by a Chi-square test, and logistic regression analysis was applied to develop a CRC diagnostic panel. To evaluate the diagnostic value of the panel, receiver operating characteristic (ROC) curve, and the area under the curve (AUC) were applied to evaluate the diagnostic value of circRNAs. The Youden index (specificity + sensitivity − 1) was used to calculate the cutoff value of the circRNAs. The statistically significant standard was P-values < 0.05. R software 3.5.1, GraphPad 7.0 and SPSS 22.0 software was used to analyze all statistical data.

## Results

### Identification of candidate circRNAs and validation

**Fig. 1:**
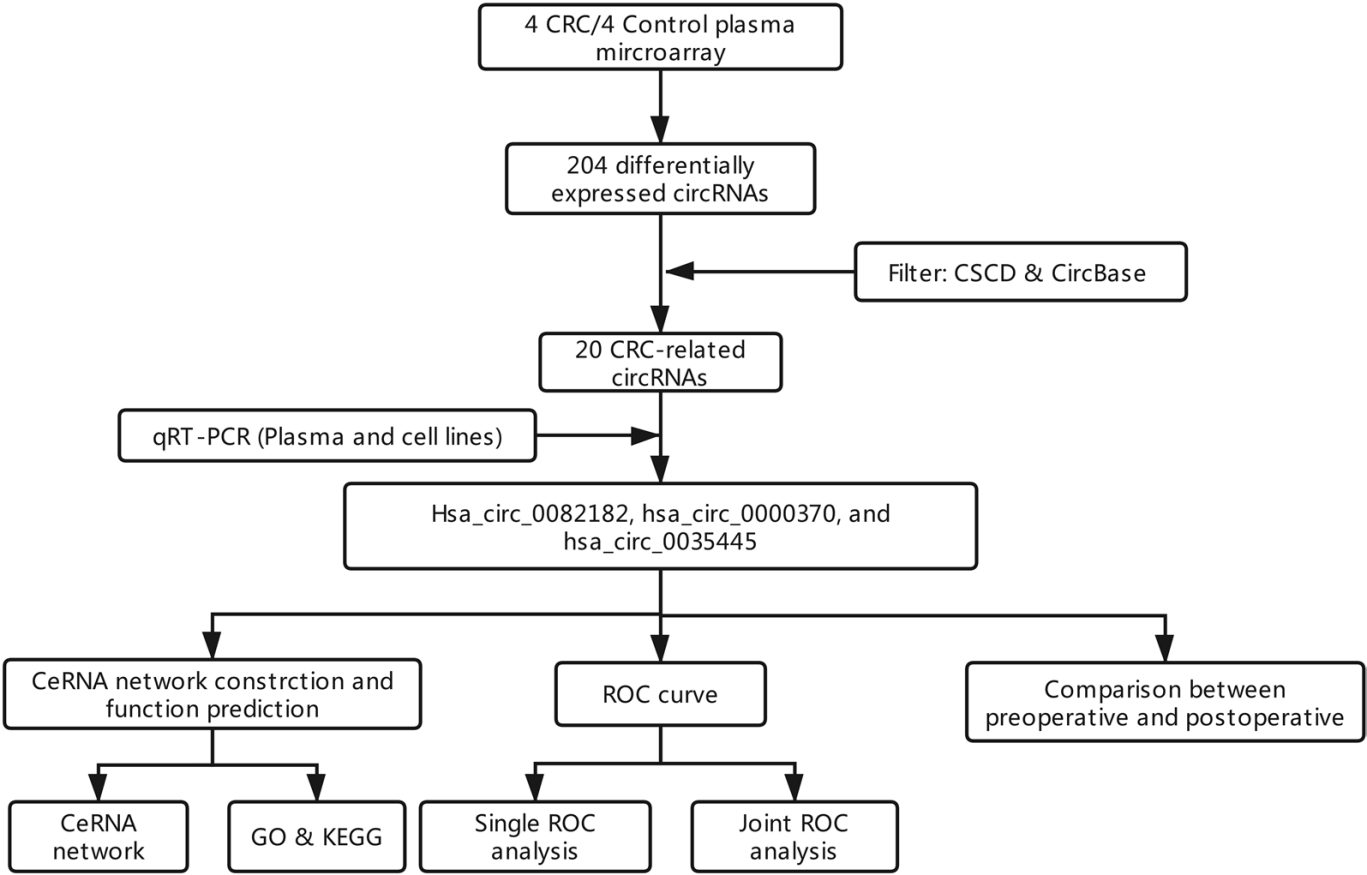
The flow chart of the whole work

**Fig. 2:**
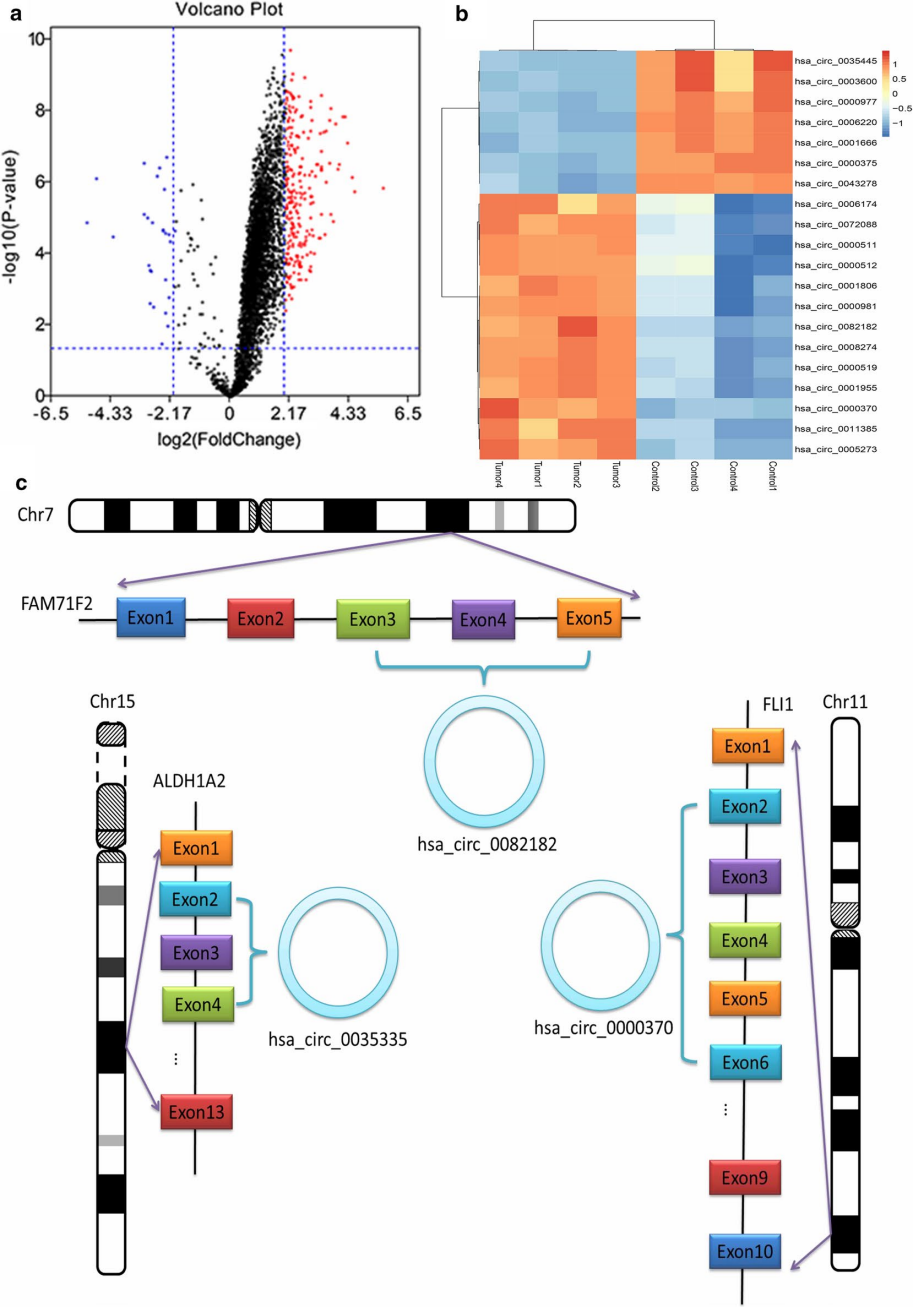
Profiling of circRNAs in the plasmas from GC patients CRC and normal plasma and the biological structure of circRNAs. **a** Volcano plot displays the circRNAs differentially expressed (fold change > 2) between CRC and normal plasma (P < 0.05). **b** Heat map shows the top 20 dysregulated circRNAs between CRC and normal plasma. **c** Schematics shows the biological structure of hsa_circ¬_0082182, hsa_circ¬_0000370, and hsa_circ¬_0035445

**Fig. 3:**
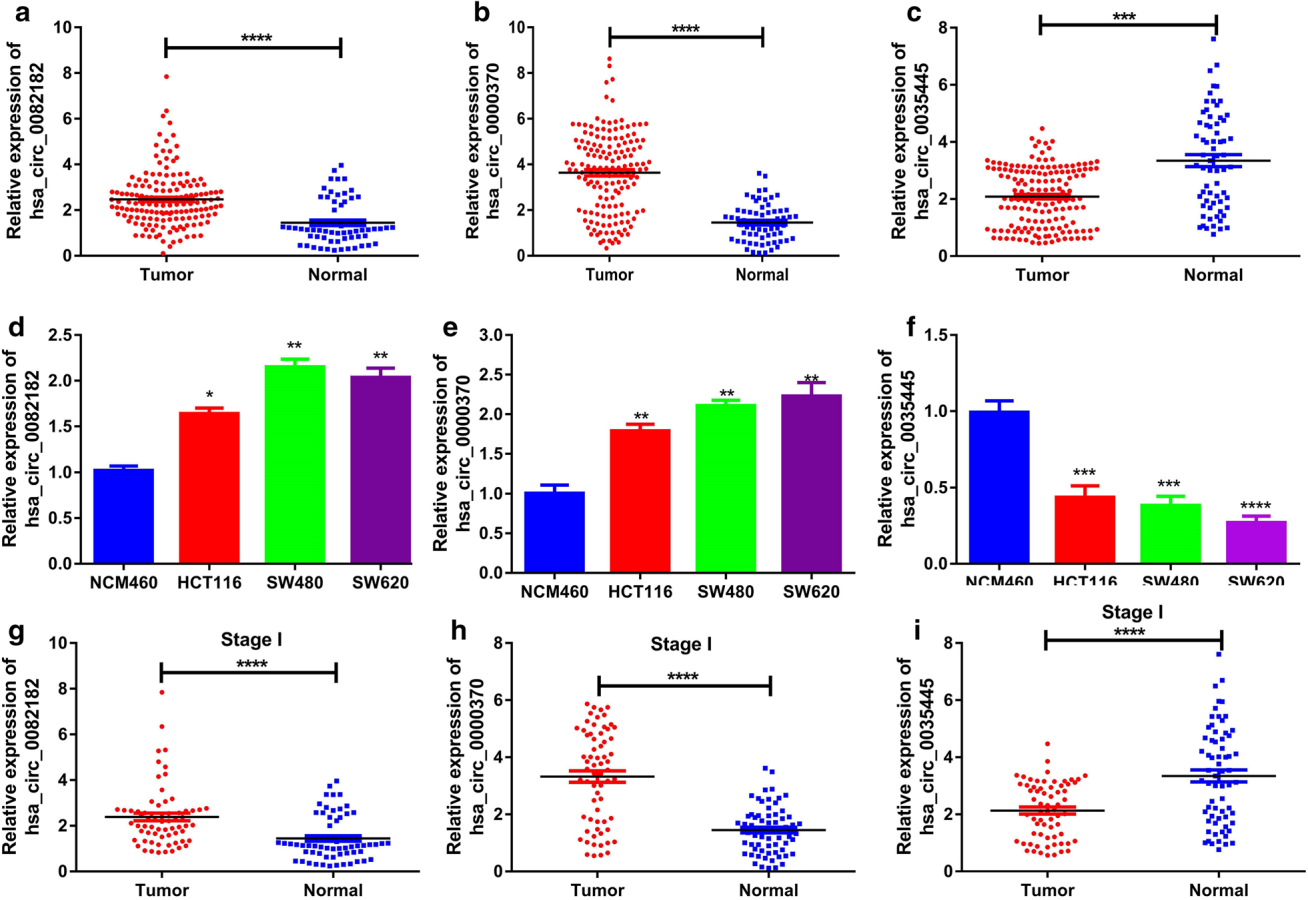
Relative expression of hsa_circ¬_0082182, hsa_circ¬_0000370, and hsa_circ-_0035445. **a**–**c** The expression of hsa_circ_0082182, hsa_circ_0000370 and hsa_circ_0035445 in CRC plasma and healthy control. **d**–**f** The expression of hsa_circ_0082182, hsa_circ_0000370 and hsa_circ_0035445 in cell lines. **g**–**i** The expression of hsa_circ_0082182, hsa_circ_0000370, and hsa_circ_0035445 in the 66 stages I-CRC plasma and normal group. Student’s t-test or ANOVA was used to compare the differences in circRNA expression between different groups. *** means *P *< 0.001, **** means *P* < 0.0001

**Fig. 4:**
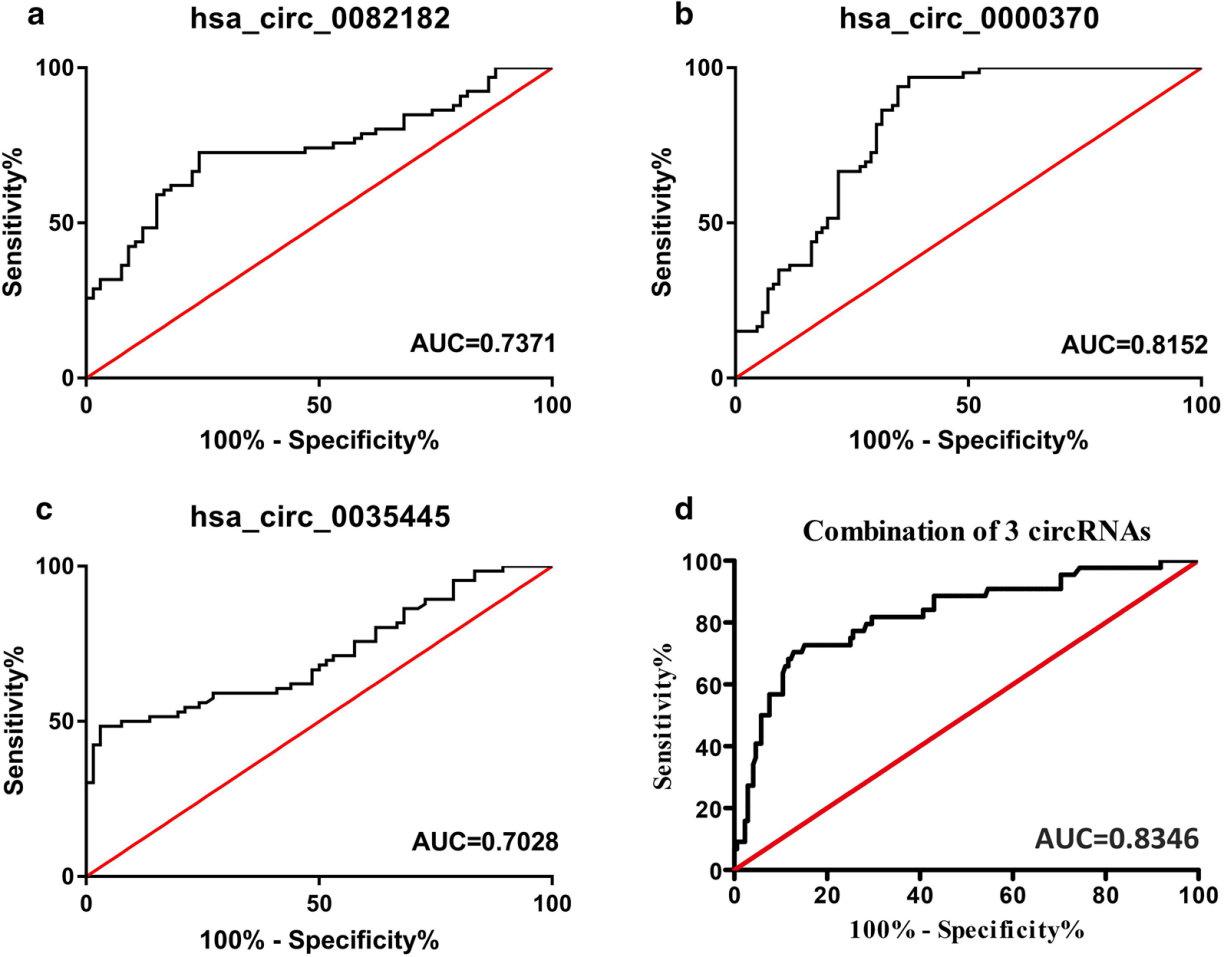
ROC curves analysis of hsa_circ_0082182, hsa_circ_0000370, hsa_circ-_0035445, and mixture of these three circRNAs (**a**–**d**)

The whole work was visualized in Fig. 1. In preliminary screenings to identify differentially expressed circRNAs in CRC plasma, we performed microarray circRNA expression analysis using eight samples, including four CRC plasma and normal plasma. As depicted by the volcano plot, our results indicated that of the 204 circRNAs differentially expressed (fold change > 2) between the CRC and normal plasma (P < 0.05), 26 were down-regulated, while 178 were up-regulated in CRC plasma (Fig. 2a). Then we used CircBase [13] and CSCD [14] database to select CRC-related circRNAs from the differentially expressed circRNAs. Among them, top 20 circRNAs were eventually identified as CRC-related circRNAs (Fig. 2b, Table 1). Then, two sets of primers, namely divergent and convergent, were designed and used. The qRT-PCR, which used 156 CRC plasma and 45 normal plasma, was performed to validate the results of microarray. Hsa_circ_0082182, hsa_circ_0000370 were shown significantly upregulated in CRC plasma, while the hsa_circ_0035445 was down-regulated (Figs. 2c, 3a–c), which was in accordance with the results of microarray analysis. In addition, the expression in cell lines revealed the same results (Fig. 3d–f). More importantly, hsa_circ_0082182, hsa_circ_0000370, and hsa_circ_0035445 also showed the same trend in the 66 stages I-CRC plasma in both CRC and normal group (Fig. 3g–i). Furthermore, ROC curve evaluation was performed to calculate the diagnostic value of three circRNAs, respectively. In Fig. 4a–c, the AUC of hsa_circ_0000370 showed the best performance in terms of its AUC was 0.8152 (95% CI 0.7647–0.8903). Hsa_circ_0082182 had an AUC of 0.7371 (95% CI 0.6807–0.8236), while the hsa_circ_0035445 had an AUC of 0.7028 (95% CI 0.6344–0.8013), respectively. It is a remarkable fact that the mixture of the three values circRNA expression of was the best discriminating evidence, with an AUC of 0.8347, highlighting their promising diagnostic performance as a panel of biomarkers for early CRC.

**Table 1:** Top 20 of differential expression circRNAs

ID	LogFC	P-value	Adj. P-value	Host gene
hsa_circ_0035445	− 6.44344	1.07E−10	2.11E−09	ALDH1A2
hsa_circ_0003600	− 5.20904	1.30E−05	3.11E−05	SOX13
hsa_circ_0000977	− 4.8718	7.68E−07	2.11E−06	NOL10
hsa_circ_0006220	− 4.27188	3.21E−05	9.38E−05	TADA2A
hsa_circ_0001666	− 2.92925	2.45E−03	7.56E−03	FAM120B
hsa_circ_0000375	− 2.5011	3.20E−02	4.30E−02	IFFO1
hsa_circ_0043278	− 2.33609	1.95E−07	6.24E−06	TADA2A
hsa_circ_0006174	5.58599	1.37E−06	2.15E−05	RAD23B
hsa_circ_0072088	4.52354	1.74E−06	2.91E−05	ZFR
hsa_circ_0000511	4.4377	7.02E−07	5.93E−07	RPPH1
hsa_circ_0000512	4.27232	7.88E−08	2.82E−06	RPPH1
hsa_circ_0001806	4.18008	1.36E−08	5.48E−06	CSPP1
hsa_circ_0000981	4.11314	1.43E−08	1.05E−04	LAPTM4A
hsa_circ_0082182	3.98254	2.96E−07	2.16E−05	FAM71F2
hsa_circ_0008274	3.92307	4.15E−07	3.22E−05	UGGT2
hsa_circ_0000519	3.81443	2.30E−08	4.19E−05	RPPH1
hsa_circ_0001955	3.74701	1.24E−05	5.18E−04	CSNK1G1
hsa_circ_0000370	3.67115	8.16E−09	5.00E−08	FLI1
hsa_circ_0011385	3.66986	7.68E−06	1.94E−05	EIF3I
hsa_circ_0005273	3.63367	2.89E−07	1.46E−06	PTK2

### Relationship between the clinicopathological characteristics and expression of the three circRNAs

**Fig. 5:**
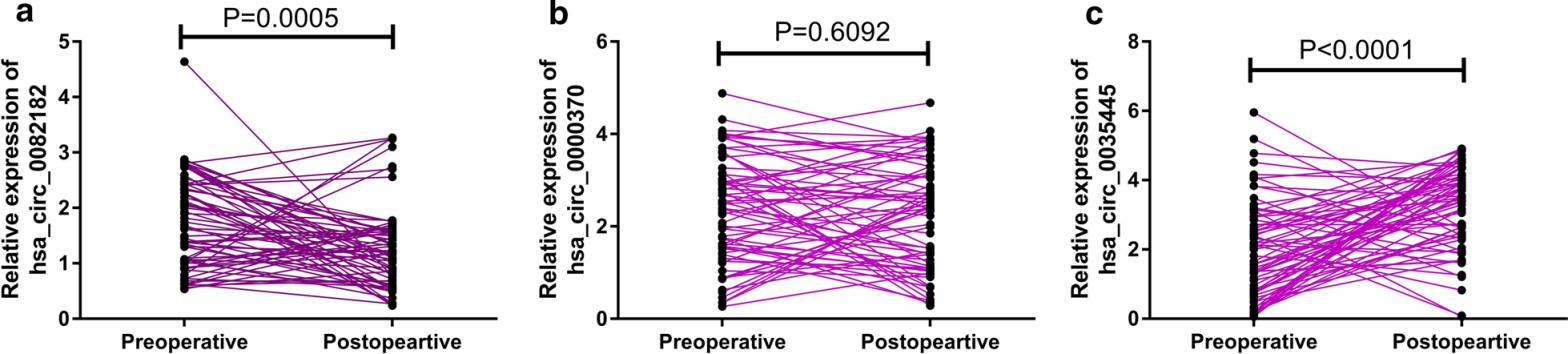
The expression of circRNAs (hsa_circ_0082182, hsa_circ_0000370, and hsa_circ_0035445) in the plasma of CRC patients before and after surgery (**a**–**c**)

A logistic regression analysis was carried out to investigate the relationships between clinicopathological characteristics and circRNAs expression. As shown in Table 2, the expression of hsa_circ_0082182 and hsa_circ_0000370 was strongly connected with lymph node metastasis, while the hsa_circ_0035445 expression was connected with the TNM stage. Notably, there is no correlation between circRNAs and ages, gender, tumor size, depth of invasion of the patient. Furthermore, we examined hsa_circ_0082182, hsa_circ_0000370 and hsa_circ_0035445 expression in the plasma of CRC patients before and after surgery. As indicated in Fig. 4a–c, hsa_circ_0082182 and hsa_circ_0035445 presented a significant difference between preoperative and postoperative stages, while hsa_circ_0000370 had no significant difference between these two stages (Fig. 5).

**Table 2:** Association between the plasma circRNA expression levels and clinicopathological characteristics of CRC

Patients	n = 156	Hsa_circ_0082182			Hsa_circ_0000370			Hsa_circ_0035445		
		Low (n = 52)	High (n = 104)	P-value	Low (n = 64)	High (n = 92)	P-value	Low (n = 113)	High (n = 43)	P-value
Age (years)				0.257			0.255			0.752
< 55	73	21	52		31	32		52	21	
≥ 55	83	31	52		33	50		61	22	
Gender				0.207			0.061			0.694
Female	91	34	57		43	48		67	24	
Male	65	18	47		21	44		46	19	
Tumor size				0.087			0.278			0.994
≤ 5	87	34	53		39	48		63	24	
> 5	69	18	51		25	44		50	19	
Depth of invasion				0.952			0.377			0.061
T1	66	22	44		29	37		53	13	
T2	48	17	31		20	28		28	20	
T3	24	8	16		11	13		19	5	
T4	18	5	13		4	14		13	5	
Lymph node metastasis				0.004**			0.014*			0.106
No	89	38	51		42	47		60	29	
Yes	67	14	53		22	55		53	14	
TNM stage				0.308			0.293			0.023*
I	75	22	53		34	41		48	27	
II and III and IV	81	30	51		30	51		65	16	

*CRC* colorectal cancer

*P < 0.05; **P < 0.01

### Prediction of the ceRNA network construction and functional enrichment analysis of targeted genes

To identify miRNAs and downstream target genes of hsa_circ-_0082182, hsa_circ-_0000370, and hsa_circ_0035445, the CircInteractome online software was applied to predict the targeted miRNA binding sites of the circRNAs. Then, the downstream mRNAs were predicted based on the TargetScan, miRTarBase and MIRDB databases. Cytoscape software was used to visualize the result (Additional file 1: Figure S1, Additional file 2: Figure S2 and Additional file 3: Figure S3). Here, we studied the downstream of circRNAs, respectively, and the targeted genes were served as input data to run a further functional analysis.

**Fig. 6:**
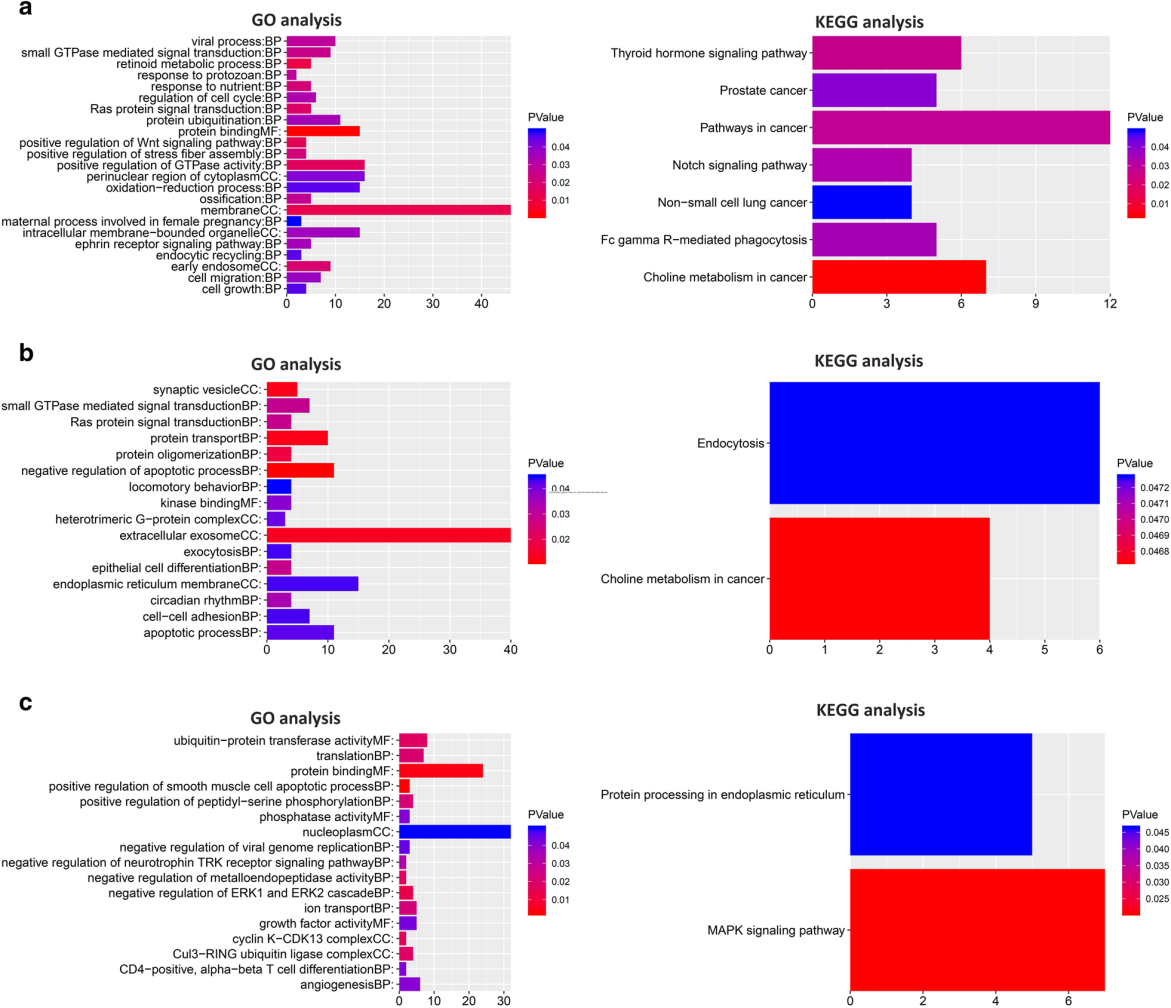
Functional analysis for three circRNAs. **a** GO and KEGG analysis of hsa_circ_0082182 based on the ceRNA network. **b** GO and KEGG analysis of hsa_circ_0000370 based on the ceRNA network. **c** GO and KEGG analysis of hsa_circ_0035445 based on the ceRNA network

Based on the DAVID database, GO and KEGG analysis were conducted for hsa_circ_0082182, hsa_circ_0000370 and hsa_0035445, respectively. As shown in Fig. 6, the targeted genes of hsa_circ_0082182 were mainly enriched in positive regulation of GTPase activity (BP), membrane (CC), protein binding (MF), and the KEGG analysis showed that the genes were mainly clustered in pathways in cancer, choline metabolism in cancer, etc. Also, we found that the targeted genes of hsa_circ_0000370 and hsa_0035445 were mainly enriched in extracellular exosome, nucleoplasm, etc., and KEGG showed that targeted genes were clustered in endocytosis, MAPK signaling pathway, etc. Some of the function or pathways, like extracellular exosome, endocytosis, were found in the functional analysis, which reflected the potential roles the three circRNAs played in CRC.

## Discussion

The initiation and development of CRC, which occurred within the colonic epithelium, could be due to the heredity and several environmental factors, especially dietary factors [15]. Since the advanced CRC patients usually have a poor prognosis, the detection of early CRC has become a huge challenge. In other words, the higher sensitive and specific biomarkers for early CRC are needed for CRC prevention and treatment [16–18]. Traditional serum biomarkers, including carcinoembryonic antigen (CEA) and CA19-9, were limited by their low sensitivity and specificity. Novel biomarkers were indeed demanded to be studied. As a type of non-coding RNA, circRNA is more stable and abundant in body fluids (including plasma, saliva and exosomes) than linear RNA and has more potential to be reliable candidates for biomarker detection [19–21]. Recently, accumulating evidence noted that circRNAs could serve as promising biomarkers for cancer diagnosis [4, 22]. Therefore, we considered that circulating circRNAs are the potential biomarkers for the early diagnosis of CRC. Non-coding RNAs were previously thought to be “transcriptional noises”, and their functions were hugely ignored [23]. CircRNAs, deriving from linear RNAs, are the exons back-splicing products [24]. As previously noted, Chen and his colleagues screened the differentially expressed circRNAs in CRC tissues and adjacent tissues. Totally 10,245 circRNAs were found to be aberrant expressed, including 3981 down-regulated and 6264 up-regulated. Functional analysis and ceRNA network were performed in their research, which provided much information for further experiments [25]. Also, Zhu et al. also investigated the different expression of circRNAs by using a circRNA microarray. They chose the most significantly up-regulated circRNA, hsa_circ_0007142, for further study and found that hsa_circ_0007142 was associated with the lymphatic metastasis and differentiation of CRC [26]. In this study, we analyzed the meaningful circRNAs in CRC plasma compared to the healthy control group by circRNA microarray, and the verified circRNAs (has_circ_0082182, has_circ_0000370, and has_circ_0035445) were chosen for further ROC curve analysis and bioinformatics analysis. After comprehensive consideration, we verified the single diagnostic value of has_circ_0082182, has_circ_0000370, and has_circ_0035445, respectively. Besides, we combined the three circRNAs expressions and interestingly found that the 3-circRNAs diagnostic panel provided the best discrimination, with higher detection accuracy than using each of them alone. Additionally, hsa_circ_0082182 and hsa_circ_0035445 showed significant differences between preoperation and postoperation stages, while hsa_circ_0000370 had no significant difference between the preoperative and postoperative stages (P = 0.6092). Since the 3-circular RNA signature was built based on the data of preoperative patients, this result had no influence on the proposed 3-circular RNA signature. To further understand the mechanisms of the above-mentioned circRNAs, we carefully searched and read the relevant studies, and found has_circ_0035445 was reported to be the highest upregulated circRNA in gastric cancer tissues [27]. So, further experiments may still need to explore the functions and mechanisms of circ_0035445 and we could find the roles it played in the digestive system. Besides, bioinformatics analysis was performed to predict the potential mechanisms of circRNAs. As shown in Fig. 6, we found some important functions or pathways, like extracellular exosomes, cell–cell adhesion (hsa_circ_0000370), positive regulation of GTPase activity, cell growth, cell migration (hsa_circ_0082182), MAPK signaling pathway (hsa_circ_0035445), etc., were cancer-related. Also, some functions, like viral process, ossification (hsa_circ_0082182), positive regulation of smooth muscle cell apoptotic process (hsa_circ_0035445) might suggest the circRNAs play roles in other diseases. Certainly, further follow-up experiments are needed for exploring its mechanism (Additional file 4: Table S1).

## Conclusions

In conclusion, differentially expressed circRNAs were found in CRC plasma by circRNA microarray analysis, and the qRT-PCR was used to validate the results. Hsa_circ_0082182, hsa_circ_0000370, and hsa_circ_0035445 were identified and ROC curves analysis was used to calculate the single and joint diagnostic value. Furthermore, we found circ_0082182 and circ_0035445 had different expression in the preoperative and postoperative plasma of LUAD patients. Finally, the bioinformatics analysis indicated that the above-mentioned circRNAs might be involved in the development of CRC.

## Supplementary information

**Additional file 1: Figure S1.** ceRNA network of has_circ_0082182.

**Additional file 2: Figure S2.** ceRNA network of has_circ_0000370.

**Additional file 3: Figure S3.** ceRNA network of has_circ_0035445.

**Additional file 4: Table S1.** Primer sequence used for qRT-PCR.

## Data Availability

The datasets used and/or analyzed during the current study are available from the corresponding author on reasonable request.

## Abbreviations

circRNAs: circular RNAs
CRC: colorectal cancer
qRT-PCR: quantitative real-time polymerase chain reaction
GO: Gene Ontology
KEGG: Kyoto Encyclopedia of Genes and Genomes
CSCD: cancer-specific circRNA-database
GAPDH: glyceraldehyde 3-phosphate dehydrogenase
STR: short tandem repeat
FBS: fetal bovine serum
ROC: receiver operating characteristic
AUC: area under the curve

## References

[1] Siegel RL, Miller KD, Fedewa SA, Ahnen DJ, Meester RG, Barzi A, Jemal A (2017). Colorectal cancer statistics, 2017. CA Cancer J Clin..

[2] Negoi I, Runcanu A, Paun S, Beuran M (2016). Right hemihepatectomy for colon cancer metachronous liver metastasis in a patient with Crohn’s disease: case report and review of the literature. Chirurgia (Bucharest, Romania: 1990).

[3] Man W, Fei Y, Wei W, Yuan Z, Wenguang C, Murugavel P, Kun W, Peifeng L (2017). Circular RNAs: a novel type of non-coding RNA and their potential implications in antiviral immunity. Int J Biol Sci.

[4] Han B, Chao J, Yao H (2018). Circular RNA and its mechanisms in disease: from the bench to the clinic. Pharmacol Ther.

[5] Li XN, Wang ZJ, Ye CX, Zhao BC, Li ZL, Yang Y (2018). RNA sequencing reveals the expression profiles of circRNA and indicates that circDDX17 acts as a tumor suppressor in colorectal cancer. J Exp Clin Cancer Res.

[6] Jin C, Wang A, Liu L (2019). Hsa_circ_0136666 promotes the proliferation and invasion of colorectal cancer through miR-136/SH2B1 axis. J Cell Physiol.

[7] Li XN, Wang ZJ, Ye CX, Zhao BC, Huang XX, Yang L (2019). Circular RNA circVAPA is up-regulated and exerts oncogenic properties by sponging miR-101 in colorectal cancer. Biomed Pharmacother.

[8] Agarwal V, Bell GW, Nam JW, Bartel DP (2015). Predicting effective microRNA target sites in mammalian mRNAs. eLife.

[9] Chou CH, Shrestha S, Yang CD, Chang NW, Huang HD (2017). MiRTarBase update 2018: a resource for experimentally validated microRNA–target interactions. Nucleic Acids Research.

[10] Hsu SD, Lin F-M, Wu W-Y, Liang C, Huang W-C, Chan W-L, Tsai W-T, Chen G-Z, Lee C-J, Chiu C-M (2011). miRTarBase: a database curates experimentally validated microRNA-target interactions. Nucleic Acids Res.

[11] Wang X (2008). miRDB: a microRNA target prediction and functional annotation database with a wiki interface. RNA.

[12] Kohl M, Wiese S, Warscheid B (2011). Cytoscape: software for visualization and analysis of biological networks. Methods Mol Biol.

[13] Glažar P, Papavasileiou P, Rajewsky N (2014). circBase: a database for circular RNAs. RNA.

[14] Xia S, Feng J, Chen K, Ma Y, Gong J, Cai F, Jin Y, Gao Y, Xia L, Chang H (2018). CSCD: a database for cancer-specific circular RNAs. Nucleic Acids Res.

[15] Gilardi L, Vadrucci M (2017). Isolated metachronous splenic metastasis from colon cancer found by 18F-FDG PET/CT. Clin Nucl Med.

[16] Melosky B (2016). Meeting an unmet need in metastatic colorectal carcinoma with regorafenib. Asia-Pac J Oncol Nurs.

[17] Łukasz P (2016). Biomarkers discovery for colorectal cancer: a review on tumor endothelial markers as perspective candidates. Dis Markers.

[18] Sekiguchi M, Matsuda T, Saito Y (2016). Surveillance after endoscopic and surgical resection of colorectal cancer. Best Pract Res Clin Gastroenterol.

[19] Li Y, Zheng Q, Bao C, Li S, Guo W, Zhao J, Chen D, Gu J, He X, Huang S (2015). Circular RNA is enriched and stable in exosomes: a promising biomarker for cancer diagnosis. Cell Res.

[20] Koh W, Pan W, Gawad C, Fan HC, Kerchner GA, Wyss-Coray T, Blumenfeld YJ, El-Sayed YY, Quake SR (2014). Noninvasive in vivo monitoring of tissue-specific global gene expression in humans. Proc Nat Acad Sci.

[21] Bahn JH, Zhang Q, Li F, Chan T-M, Lin X, Kim Y, Wong DTW, Xiao X (2015). The landscape of MicroRNA, Piwi–interacting RNA, and circular RNA in human saliva. Clin Chem.

[22] Bach DH, Lee SK, Sood AK (2019). Circular RNAs in cancer. Mol Ther Nucleic acids.

[23] Mattick JS, Makunin IV (2006). Non-coding RNA. Hum Mol Genet.

[24] Beermann J, Piccoli M-T, Viereck J, Thum T (2016). Non-coding RNAs in development and disease: background, mechanisms, and therapeutic approaches. Physiol Rev.

[25] Chen S, Zhang L, Su Y, Zhang X (2018). Screening potential biomarkers for colorectal cancer based on circular RNA chips. Oncol Rep.

[26] Zhu CL, Sha X, Wang Y, Li J, Zhang MY, Guo ZY, Sun SA, He JD. Circular RNA hsa_circ_0007142 is upregulated and targets miR-103a-2-5p in colorectal cancer. 2019, 2019:9836819. 10.1155/2019/9836819 PMC6617873 31346335

[27] Shao Y, Li J, Lu R, Li T, Yang Y, Xiao B, Guo J (2017). Global circular RNA expression profile of human gastric cancer and its clinical significance. Cancer Med.

