# Supplementary figures and images for: A 3-circular RNA signature as a noninvasive biomarker for diagnosis of colorectal cancer
Source: Cancer Cell Int. 2019 Nov 4;19:276. doi: 10.1186/s12935-019-0995-7 (PMC6829842; doi:10.1186/s12935-019-0995-7)

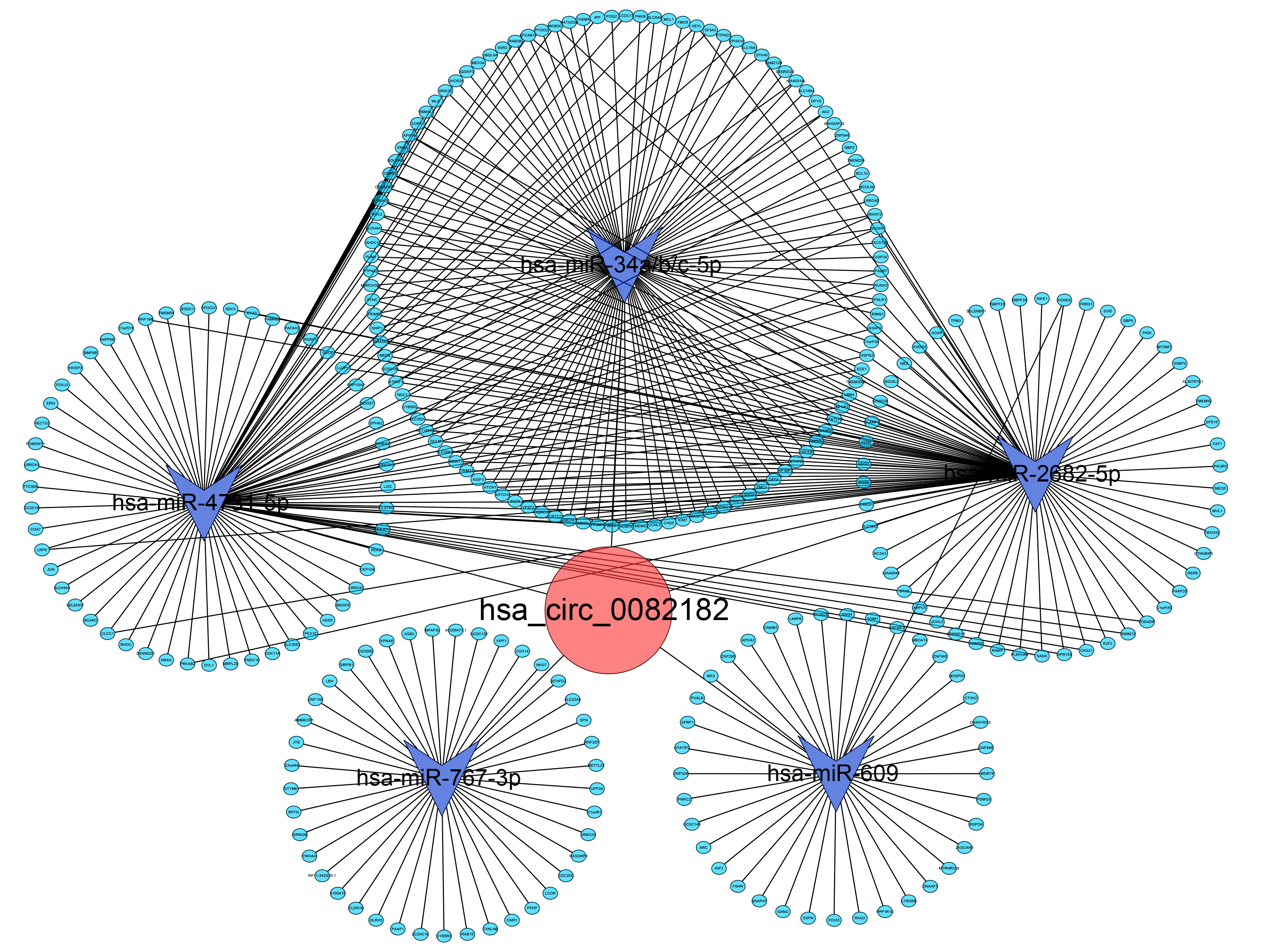

Supplement: Supplementary file 1 — Additional file 1: Figure S1. ceRNA network of has_circ_0082182. [file 12935_2019_995_MOESM1_ESM.jpg]

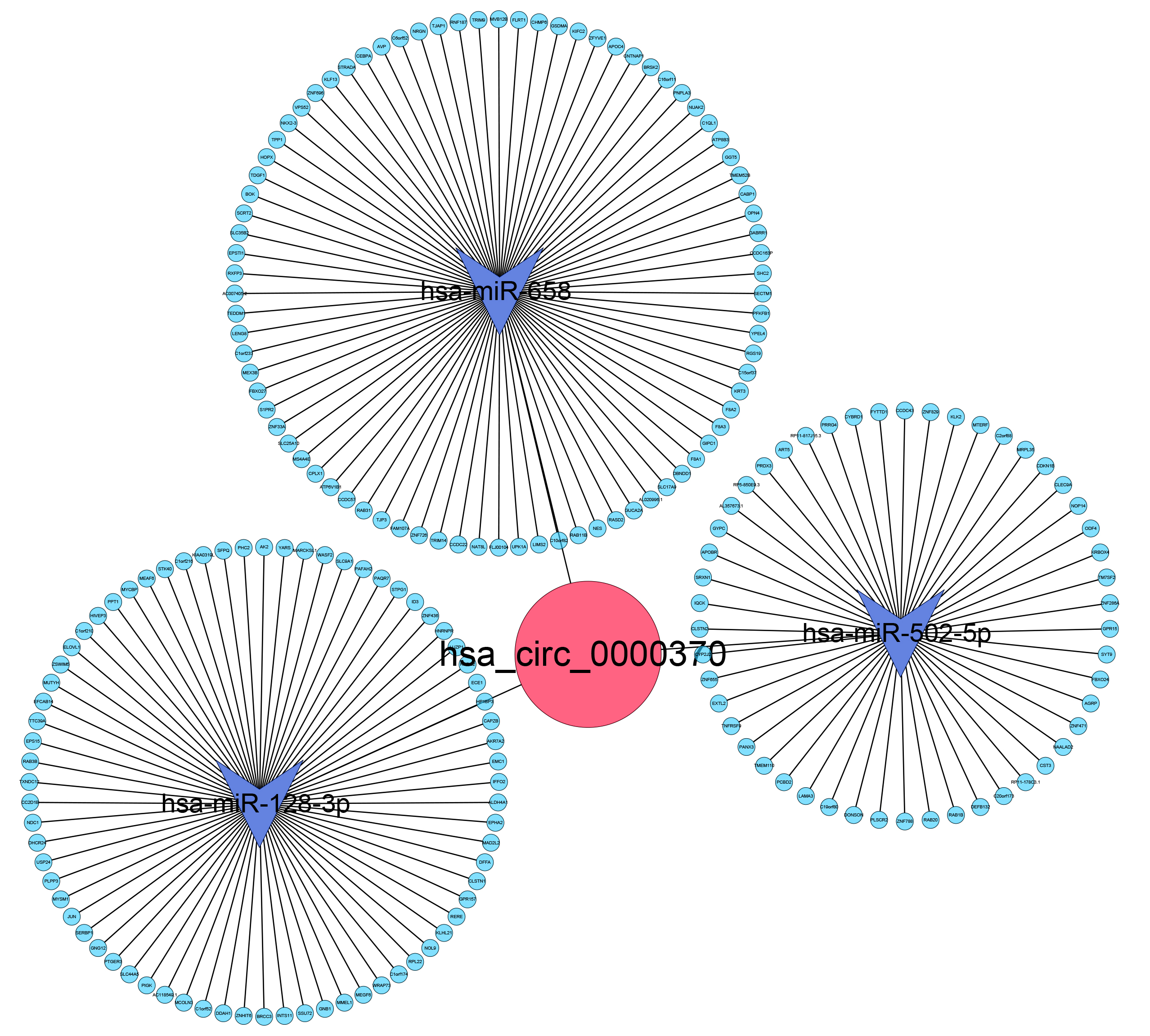

Supplement: Supplementary file 2 — Additional file 2: Figure S2. ceRNA network of has_circ_0000370. [file 12935_2019_995_MOESM2_ESM.jpg]

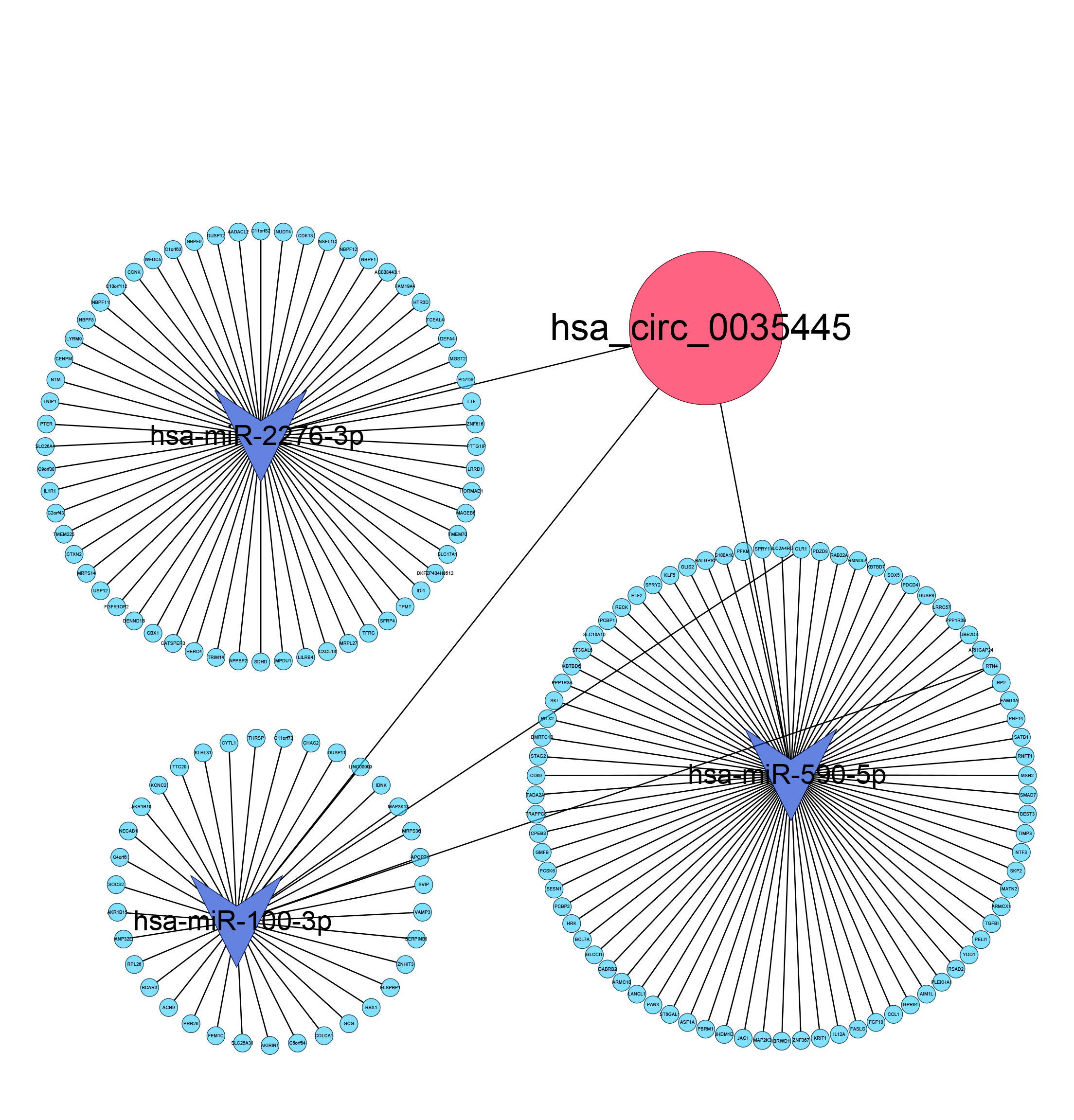

Supplement: Supplementary file 3 — Additional file 3: Figure S3. ceRNA network of has_circ_0035445. [file 12935_2019_995_MOESM3_ESM.jpg]
