# Supplementary material for: A 3-circular RNA signature as a noninvasive biomarker for diagnosis of colorectal cancer
Source: Cancer Cell Int. 2019 Nov 4;19:276. doi: 10.1186/s12935-019-0995-7 (PMC6829842; doi:10.1186/s12935-019-0995-7)
Supplement: Supplementary file 4 — Additional file 4: Table S1. Primer sequence used for qRT-PCR. [file 12935_2019_995_MOESM4_ESM.docx]

**Table S1. Primer sequence used for qRT-PCR.**

| **ID** | **Primer sequence** |
| --- | --- |
| hsa_circ_0082182 | F:TATAATGCTCCTTTTCCACTGTGAGC |
|  | R:TTTTCGGTCACTGTCCTAACCC |
| hsa_circ_0000370 | F:ATGGATGGCAAGGAACTGTGTAA |
|  | R:TCAAAGAGGGACTGGTCGTC |
| hsa_circ_0035445 | F:TACGCAGGCTGGGCTGATAA |
|  | R:ACACTGAACCAAGAGAGAAAGCC |
| GAPDH | F:CTGACTTCAACAGCGACACC |
|  | R:TGCTGTAGCCAAATTCGTTGT |
